# Spin Coating and Micro-Patterning Optimization of Composite Thin Films Based on PVDF

**DOI:** 10.3390/ma13061342

**Published:** 2020-03-16

**Authors:** Anh Ngoc Nguyen, Jeanne Solard, Huyen Thi Thanh Nong, Chirine Ben Osman, Andres Gomez, Valérie Bockelée, Sylvie Tencé-Girault, Frédéric Schoenstein, Maite Simón-Sorbed, Anna Esther Carrillo, Silvana Mercone

**Affiliations:** 1 Laboratoire de Sciences des Procédés et des Matériaux (LSPM-CNRS UPR-3407), Université Sorbonne Paris Nord (USPN), 93430 Villetaneuse, France; anhnn@hus.edu.vn (A.N.N.); thanhhuyen.vltn@gmail.com (H.T.T.N.); valerie.bockelee@lspm.cnrs.fr (V.B.); frederic.schoenstein@univ-paris13.fr (F.S.); 2 Institute of Materials Science, Vietnam Academy of Science and Technology, Cau Giay Distr., Hanoi, Vietnam; 3 Laboratoire de Physique des Lasers (LPL-CNRS UMR-7538), Université Sorbonne Paris Nord (USPN), 93430 Villetaneuse, France; jeanne.solard@univ-paris13.fr; 4 Institute Jean Lamour, UMR 7198 CNRS - Lorraine University Campus Artem, 54000 Nancy, France; 5 R&I Silica Synthesis Engineer, SOLVAY, 92400 Courbevoie, France; ch.benosman@gmail.com; 6 Instituto de Ciencia de Materiales de Barcelona (ICMAB-CSIC), Campus UAB, 08193 Bellaterra, Spain; agomez@icmab.es (A.G.); msimon@icmab.es (M.S.-S.); anaesther@icmab.es (A.E.C.); 7 PIMM, Arts et Metiers Institute of Technology, CNRS, Cnam, HESAM University, 151 Boulevard de l’Hopital, 75013 Paris, France; Sylvie.GIRAULT@ensam.eu

**Keywords:** PVDF, spin coating, magnetic nanoparticles, composite thin films

## Abstract

We optimize the elaboration of very thin film of poly(vinylidene fluoride) (PVDF) polymer presenting a well-controlled thickness, roughness, and nano-inclusions amount. We focused our effort on the spin coating elaboration technique which is easy to transfer to an industrial process. We show that it is possible to obtain continuous and smooth thin films with mean thicknesses of 90 nm by properly adjusting the concentration and the viscosity of the PVDF solution as well as the spin rate and the substrate temperature of the elaboration process. The electro-active phase content versus the magnetic and structural properties of the composite films is reported and fully discussed. Last but not least, micro-patterning optical lithography combined with plasma etching has been used to obtain well-defined one-dimensional micro-stripes as well as squared-rings, demonstrating the easy-to-transfer silicon technology to polymer-based devices.

## 1. Introduction

Piezoelectricity allows bidirectional conversion between electric voltage and mechanical stress, providing not only excellent electromechanical conversion but also strong spontaneous polarization. Indeed, ferroelectric compounds show good piezoelectric performances (i.e., high piezoelectric coefficient). In a similar manner, magnetostriction is well known to allow inter-conversion between mechanical stress and magnetization. The most common magnetostrictive materials are based on iron, which present strong magnetic polarization (i.e., ferromagnetic properties). Judiciously combining these two kinds of materials, it is possible to obtain magnetoelectric (ME) composites simultaneously exhibiting ferroelectric/piezoelectric and magnetostrictive/ferromagnetic properties. These hybrid systems are promising candidates for the development of magnetic field sensors thanks to their large strain-mediated ME coupling with room temperature operation [1,2]. The magnetic/electric field dependence of the ME coefficient is determined by the good coupling between the piezoelectric (or electrostrictive) and the magnetostrictive phase as well as by the strain transfer efficiency between them. This latter strongly depends, at its turn, on the connectivity and the microstructure of the two phases. In this frame, although ceramic-based ME composites have been intensively investigated, they suffer from a main drawback as they usually present high dielectric losses that prevents them to be suitable for a new generation of device. Also, this latter is required to be thin, soft, lightweight, flexible and also bio-friendly for a wide variety of important applications, such as microrobotics, flexible/wearable devices, and bio-implanted sensors [3,4,5,6]. All these requirements impose challenges for conventional piezoelectric ceramics, which needs high processing temperatures, have almost no mechanical flexibility and contain potential toxic elements. A way to overcome this problem is to use polymer based ME composites that can provide colossal ME response due to a large piezoelectric stress coefficient and a great displacement transfer capability of the polymer [1,2,7,8,9,10,11,12,13,14]. Recent developments on polymer processing [15] show the possibility of obtaining high dielectric constant and low dielectric loss by easy fabrication strategies. Besides, the potential of organic–inorganic materials as new smart compounds for the future nanoelectronics is, without any doubt, linked to the fact that they are able to combine, at a nanoscale level, the benefits of the organic phase with the advantageous characteristics of crystalline inorganic solids. In order to emphasize this ME effect a special configuration named “0–3” is recommended [1,9,10]. This kind of composite is artificially obtained by magnetic nanoparticles (NPs) (0 dimensional), embedded in a piezoelectric polymer (3-dimensional). They present several advantages: ease of process, malleability (even non-flat) configurations, light weight, low cost and scalable production methods compatible with industrial requirements for flexible structures [16,17,18,19]. Among organic piezoelectric materials, Polyvinylidene fluoride (PVDF) (β-phase) polymer has been largely studied as it presents a good piezoelectric coefficient than that observed in any other polymer (piezoelectric coefficient (d_33_) has been found for the best cases around −30 pC/N and for worst cases around −6 pC/N), also excellent mechanical flexibility as well as low production cost compared to ceramics. Thus it is a good candidate for developing flexible multifunctional composites for the electric control of the magnetic properties. Depending on the crystallization conditions, electrical poling, and mechanical drawing, PVDF can crystallize in four different phases, called α, β, γ, and δ [20]. Only the β and γ phases are electro-active and thus present the suitable piezoelectric properties. Thus, the improvement of these latter phases content in a PVDF-based structure is highly recommended for the applications. There are variety of strategies to achieve the optimization of high polar content, like using mechanical stretching [18], applying a high electrical field during the elaboration [19], blending with polymethylmethacrylate (PMMA) homopolymer [21] or with PMMA-based block copolymer [22], doping the PVDF polymer matrix by inorganic nano-fillers [23,24] and by functionalizing these latter grafting specific molecules before their inclusion in the PVDF matrix [25,26]. However, using mechanical stretching and applying a high electrical field are only suitable for preparing self-standing films with thickness in a range of several µm and thus not suitable for standard Si-based technology. On the contrary, using nano-inclusions favors the crystallization of the polar (β) phase of the PVDF [23], which in principle may increase the piezoelectric macroscopic response without preventing the elaboration of thin films on conductive substrate. The efficiency of this latter as well as the mechanical properties of the PVDF/ nano-fillers composite films should also be related to the uniform dispersion of the nano-inclusions [24,27,28] and thus the optimization of the organic/inorganic interface (i.e., functionalization process). As a matter of fact, there are several studies in literature focusing on the elaboration of PVDF films and debating on which methods favor the formation of the polar phases. Only two elaboration methods seem to be promising: Langmuir–Blodgett (LB) [29] and spin coating [30]. Unfortunately, the first technique is a time-consuming one and complicated to perform [31] with a low efficiency in reproducibility. Additionally, this technique is not really adapted to the PVDF thin film elaboration due to the high hydrophobicity of this latter [25,32]. For these reasons, the spin coating method has lately been gathering more attention because of its many advantages compared to the LB one. Spin coating is fast and easy to operate and it allows producing a smooth and uniform thin film with desired and well-controlled thickness without mentioning its high reproducibility. Besides, the operating system is very cheap and easy to find in the standard clean room where the devices are elaborated. Hence, it is very easy to transfer to the technological industry [30]. Furthermore, spin coating technique can be easily adapted to the elaboration of PVDF/nano-fillers composite thin film, due to the possibility of dispersing the nano-fillers in the polymer solution before the elaboration process. Despite all these benefits, to our knowledge, only few works have reported on the spin coating elaboration of the PVDF system and even less on the composite film based on it. Most of them focus on very thick film (more than 1 μm of thickness) presenting problems of porosity and homogeneity of surface. For instance, Ramasundaram et al. [30] reported that the crystallization of PVDF strongly depends on the substrate temperature during the spin coating. Their results show that a homogeneous film showing very high roughness value (~70 nm) and high β phase content can be obtained by controlling the heat supply during the spin coating. PVDF crystallizes into the β phase at 40, 50, 60, and 70 °C, while at the ambient temperature of 20 °C and 30 °C it crystallizes into the non-electroactive α phase. Abdullah et al. [33] studied the influence of various substrates on the phase and morphology of PVDF film. The results show that the ITO substrate favors the β phase crystallization more than silicon, glass, and silver coated glass substrate and the β phase content measured is of 82.3%. However, the film thickness is high (i.e., 20 µm) with high porosity (i.e., non-continuous film) and roughness. These authors also reported that the porosity of the film increases with increasing spinning rate as well as the agglomeration of the polymer on the surface of the substrate. On the contrary, at low spinning rate (500 rpm) the chain-folded fiber-type morphology, called spherulites [19,20,21,22,25,31], is observed by a surface analysis and it is attributed to the β phase. The influence of thermal annealing on the morphology of the spin-coated films was studied by Cardoso et al. [34] and showed that the low evaporation rate of solution yields to the formation of pores. To compensate this effect, the thin film was annealed at a fixed temperature between 20 °C and 80 °C after deposition. The results indicated that the higher the thermal annealing is, the less porous the structure is and the less β phase is crystallized. The higher the spin rate is, the lower the viscosity is and the thinner the obtained film is. A smooth and flat film of ~4 µm thick with 75% of β phase content has been obtained by annealing at 80 °C [35]. Li et al. [5] reported that the thickness and roughness of the film also strongly depend on the relative humidity and the substrate temperature during the elaboration. Both thickness and roughness increase with increasing humidity and decrease with the increasing substrate temperature. In this case, the film obtained has a thickness of 1 µm and the roughness is less than 10 nm when the 0% of humidity is reached and a high substrate temperature is used [5]. He et al. [36] show that adding Mg(NO_3_)_2_ to the PVDF solution and drying at 100 °C the as-deposited film, it can obtain a hybrid film of 1 µm thick with dominant β phase. Ourry et al. [37] prepared a self-standing composite PVDF-CoFe_2_O_4_ with the size of nanoparticles (NPs) smaller than 10 nm. Their results showed that the size and the amount of NPs affect the β phase content of the hybrid micrometric self-standing film. The higher percentage of polar PVDF phase was found in the sample processed at 80 °C for the higher amount of NPs. The morphology showed two different spherulitic morphologies: the granular structure corresponding to α PVDF and the big fiber-type spherulites corresponding to β PVDF [37]. All these results bring up the question of whether or not it is possible to realize by an easy-to-transfer technique both neat PVDF thin films as well as the hybrid ones showing low roughness (lower than 10 nm), thickness smaller than 100 nm that is homogeneous over a large surface on silicon-based conductive substrates. All these characteristics are mandatory in order to probe in the near future the possibility of using these polymer-based films for the next generation of multifunctional (i.e., magnetoelectric) microelectronics based on the standard silicon technology. In this context, we have therefore investigated the optimization of very thin films (thickness less than 100 nm) by spin coating technique. We analyzed the influence of external parameter acting during the elaboration on the promotion of the electro-active phase of the PVDF and on the optimization of the hybrid inorganic and organic interface for the composite films. We reported on the electro-active phase content evolution of the PVDF films as function of the elaboration parameters, as well as on the magnetic properties of the nano-inclusions. After the process optimization, we also demonstrate the easy feasibility of PVDF micro-lines as well as squared-rings based structures by standard silicon lithography technique. This open the way for the easy use of PVDF thin film in future magnetoelectric devices.

## 2. Materials and Methods

**Figure 1:**
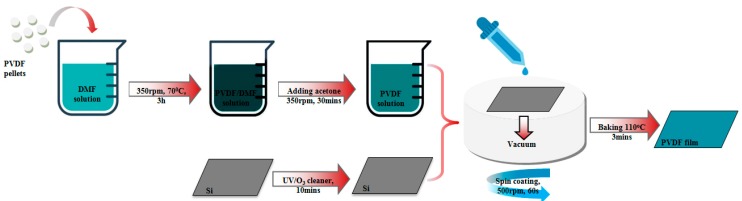
Scheme of the PVDF thin film elaboration steps.

All the chemicals used were analytical grade and used as purchased from Sigma-Aldrich Company. The poly (vinylidene fluoride) (PVDF) solution was prepared with PVDF pellets (M_W_ = 180,000 g·mol^−1^) dissolved totally in N-N dimethyl formamide (DMF) during 3 h at 70 °C under mechanical stirring. The concentration of PVDF in solution was kept constant at 2 wt % concentration during all our study. For higher concentration, PVDF pellets were not well-dissolved and presented agglomerates of PVDF on the film surface. For lower concentration, the films were not homogeneous. The process of spin coating was carried in two steps: a first step at low speed (500 rpm) to spread the PVDF solution over the substrate and a second step to have a uniform thin film at the desired thickness. The acceleration of the first step varied from 50 rpm s^−1^ to 500 rpm s^−1^. Acetone was added to the solution in order to study the effect of the evaporation rate. PVDF films were deposited by spin coating (Eberle) on different type of substrate: glass substrate, Silicon substrate (Silchem Handelsgesellschaft mbH) and silicon coated with 100 nm of aluminum by evaporation process. All the substrates were pretreated before PVDF deposition in order to remove all the contaminants (see Figure 1): firstly, substrates were ultra-sonicated in acetone during 5 min, then in isopropanol during 5 min to remove organic contaminants, then they were ultra-sonicated in distilled water during 5 min to remove the inorganic contaminants together with dust and small-unwished particles and finally the substrates were dried under nitrogen gas. After the deposition process, the films were dried at 110 °C during 3 min in an oven. 

PVDF composite films were produced with a similar approach (see Figure 1). The specific amount of NPs was added into the PVDF solution, which was put under ultrasonication for 1 h (see Section S4 of Supplementary Materials for more details on the spin coating set up and used concentrations). The deposition process was the same as described for PVDF solution. Nano-inclusions (i.e., isotropic nanoparticles of Ni_0.5_Zn_0.5_Fe_2_O_4_) were obtained by polyol synthesis method [38,39,40,41,42,43] and present an average diameter of 5 nm (see Supplementary Materials for NPs synthesis details and XRD analysis). Their magnetic properties have been characterized before and after their inclusion into the polymer solution in order to verify the impact of the elaboration process and study the organic/inorganic interface effect on their magnetic behavior. Micro-structuration of the PVDF thin films has been obtained using mask aligner (MJB4 Suss Microtech, Andover, UK) combined with reactive ion etching (RIE) system (200 IL Dry etcher, Corial, Bernin, France). Photoresist AZ1505 (Microchemicals, Ulm, Germany) was used as a mask during etching process in trifluoromethane (CHF_3_) plasma. PVDF film thickness was measured by profilometer (Alphastep IQ, Klatencor, Milpitas, California). The surface structures and film roughnesses were analyzed by statistical studies of high-resolution AFM images (AFM, Bruker D3100, Bruker France, Palaiseau, France). The analysis is reported in Appendix A. X-Ray diffraction (XRD Equinox 1000, ThermoFisher Scientific, Karlsruhe, Germany) was performed at room temperature with a Cu-target Kα radiation (λ = 1.54056 Å). InfraRed (IR) spectroscopy (Thermo Nicolet, AVATAR 370 FTIR, ThermoFisher Scientific, Karlsruhe, Germany) was carried out over a range of 600–1500 cm^−1^ in ATR mode. Magnetization as function of the magnetic field (Quantum Design MPMS 3 magnetometer, Quantum Design Inc., San Diego, CA, USA) was collected in the magnetic field range of [−1 T, +1 T] at 300 K. AC susceptibility at different frequency in the range of 10 Hz to 1000 Hz, have been performed under a magnetic field of 0.1 Tesla as function of temperature (10 K to 100 K).

## 3. Results

The main part of our work will focus on the optimization process of the spin coating technique parameter allowing the elaboration of thin films of thicknesses lower than 100 nm, continuous, and smooth thus recovering all the substrate surface (4 × 4 mm) and presenting a mean roughness lower than 10 nm. In this purpose we analyze firstly, by atomic force microscopy (AFM), the surface of our films as function of the elaboration parameters (Section 3.1). We studied then the electro-active phase content of our samples by XRD and IR spectroscopies (Section 3.2 and Section 3.3). We show, in the case of the composite films, the good properties of the magnetic nano-inclusions (Section 3.4). Finally, we show the good resolution of micro-lines and squared-rings (2 micron of diameter) obtained by a standard structuration technique (Section 3.5).

### 3.1. AFM Images Results

**Figure 2:**
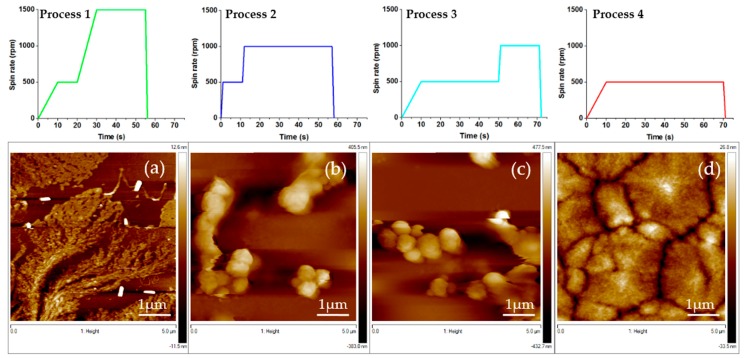
5 × 5 µm AFM micrographs showing the morphology of PVDF spin-coated thin films with the corresponding spin coating processes on the top. Bottom: thin film obtained (**a**) by process 1; (**b**) by process 2; (**c**) by process 3; (**d**) by process 4.

The influence of different elaboration parameters on the morphology and surface quality of the PVDF films are studied here. Our aim is to obtain very thin layer of PVDF polymer with a thickness lower than 100 nm presenting a smooth surface (typically roughness values requested are lower than 10 nm). The films have to be smooth, flat, and continuous over a large surface to be suitable for the microstructuration process in view of industrial microelectronics applications. To this purpose, the first investigated elaboration parameter for the spin coating process has been the speed rate. Four processes have been studied (see Figure 2). As a result, smooth and continuous thin film was obtained with low acceleration rate (Figure 2d) whereas too high acceleration rate and rotation rate led to non-continuous thin films with agglomerates of PVDF (Figure 2a–c). Again, best results of continuous and homogeneous thin film were obtained with the lowest speed rate of 500 rpm (see Figure 2d). Higher speed rates lead to non-homogeneous films (Figure 2a–c).

**Figure 3:**
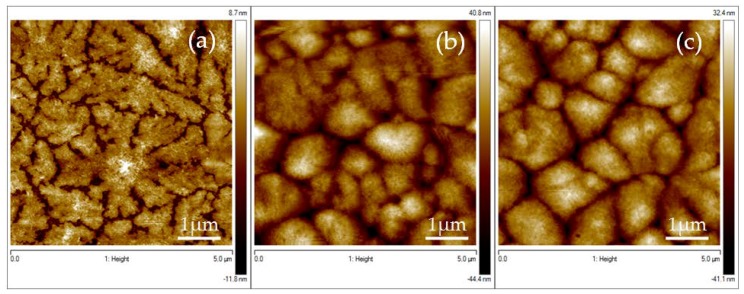
5 × 5 µm AFM micrographs showing the morphology AFM images of PVDF thin films with different volume ratio of DMF/acetone in the solution (**a**) 1:1 (**b**) 1:2 (**c**) 1:3.

This first study for the optimization of the optimal spin-rate process allows us to conclude that thanks to a 2 steps process is possible to obtain continuous and thin PVDF films. Firstly, the spin rate has been increased from 0 to 500 rpm within 10 s. Then, it has been kept at 500 rpm during 60 s and then reduced to zero in 1 s (process number 4, Figure 2d). From now on, this is the process used for the elaborated samples. In order to improve the quality of the PVDF film, the effect of the DMF-acetone ratio of the solvent was studied by changing the volume fraction of acetone in the solution (Figure 3). Indeed, acetone is a volatile solvent that present a lower dynamic viscosity than DMF one (respectively 0.33 mPa·s compared to 0.92 mPa·s at 20 °C). Volume ratio of DMF/acetone was varied from 1:1 to 1:3 in order to increase the fraction of the lower viscous fluid in our solution. Results are summarized in Table 1. The addition of acetone in the solution improves the quality of the PVDF thin film. With a volume ratio of 1:3, a continuous thin film is obtained with mean roughness of 10 nm and thickness of 90 nm (Figure 3c) (see Appendix A for details on the roughness analysis).

**Figure 4:**
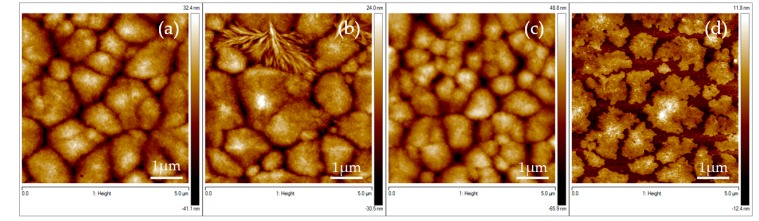
5 × 5 µm AFM micrographs showing the morphology of PVDF thin films spin-coated on silicon at different temperature: PVDF films obtained at (**a**) 20 °C; (**b**) 40 °C; (**c**) 60 °C and (**d**) 80 °C.

It is worth mentioning here that using conductive substrates in order to elaborate our thin films is mandatory in order to benefit of the PVDF piezoelectric properties [44,45]. Thus, we transferred the optimized process (spin rate and DMF/acetone ratio parameters) on different type of substrates: doped silicon conducting substrates and silicon coated by conductive aluminum. The results reported in Table 1 are maintained for both type of substrates (see also Table 2) and demonstrate that the process is highly transferable with the required characteristics on both substrates. In order to go further in the optimization of the PVDF thin film elaboration, we decided also to study the effect of the substrate temperature. There is a common agreement in literature [30] on the fact that increasing the substrate temperature during the deposition process facilitates the crystallization of the electro-active β-phase content in the PVDF system. This should promote the piezoelectric characteristics of the films which is a major goal of our elaboration optimization study. We varied the substrate temperature from room temperature (RT) up to 80 °C by heating the substrate in an oven during 10 min just before the spin coating process (see Section S4 of the Supplementary Materials for more details on the heating process). Topography results in Figure 4 clearly show granular and spherulitic structures simultaneously with lamellae ones (Figure 4b) typical of the PVDF α and β phases. When the substrate temperature increases, the formation of spherulitic structures increases as well as their characteristical sizes. At high temperature (80 °C), the PVDF film becomes not homogeneous (Figure 4d) due to the quick acetone evaporation process. AFM images in Figure 4 have been analyzed and their morphology characterics are summarized in Table 2 as function of the substrate temperature and the substrate type (see Appendix A for details on the roughness analysis).

**Figure 5:**
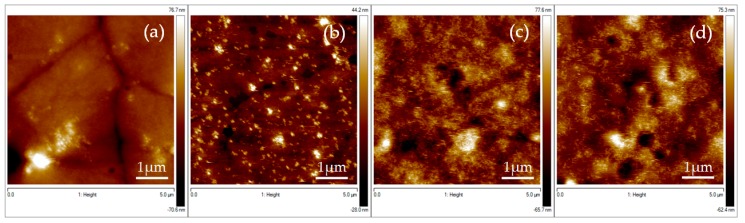
5 × 5 µm AFM micrographs showing the morphology of the composite PVDF thin film on silicon substrate with different concentrations of NPs (**a**) 0.5 wt % of non-functionalized NPs; (**b**) 0.5 wt % of functionalized NPs; (**c**) 1 wt % of non-functionalized NPs; and (**d**) 1 wt % of functionalized NPs.

Finally, magnetic nano-inclusions were added to the PVDF solution before the spin coating process. The idea was to create artificial coupling between the electro-active phase of the organic thin film and the standard inorganic ferromagnetic nanoparticles with the aim of generating an artificial magnetoelectric material with concomitant ferroelectric/piezoelectric and ferromagnetic/magnetostrictive properties coexisting at room temperature. In order to improve the dispersion of the NPs, and thus the quality of the interface between the NPs and the polymer, the NPs were functionalized before being dispersed into the PVDF solution. As a matter of fact, ferromagnetic nanoparticles tend to easily agglomerate due to their strong magnetic attraction. In order to avoid their agglomeration as much as possible, a possibility is to functionalize them with specific molecules and a well-defined procedure [25,26] (see Supplementary Materials, Section S3 for more details on the functionalization process). In this case, we used the optimized spin coating process at room temperature on silicon wafer, while the percentage of NPs and their functionalization were varied. Figure 5a,b shows the surface of composite PVDF films with 0.5 wt % of NPs respectively non-functionalized and functionalized. Figure 5c,d shows the surface of composite PVDF films with 1 wt % of NPs respectively non-functionalized and functionalized. The films are smooth and present a roughness value (Table 3) which increases, as expected, with the increase of the NP amount (Table 3).

### 3.2. X-Ray Diffraction Data of Neat and Composite PVDF Films on Different Substrates

**Figure 6:**
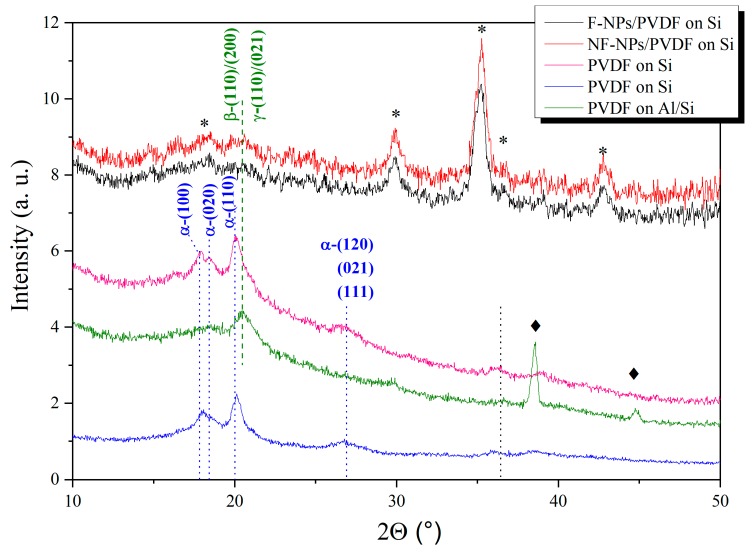
X-ray diffraction patterns acquired on several produced samples of neat PVDF thin films and composite ones. Details of different elaboration conditions of the films are reported in the main text. The main Bragg peaks of the PVDF α and β crystalline phases are indexed, as well as the diffraction lines associated with the crystalline spinel nanoparticles (∗) and with the aluminum (♦).

Figure 6 shows the XRD pattern of several produced samples. It is well-known that PVDF can crystallize in four different phases depending on the elaboration conditions, electrical poling and mechanical drawing. These phases are called: α, β, γ, and δ. The most commonly crystallized phase is the α one, which is non-electro-active. β and γ are more delicate to obtain, but also the more electro-active one with the β phase presenting the best piezoelectric properties [20]. Our optimization process has to take into account elaboration parameters that promote the amount of this phase compared to the others in the PVDF films. In Figure 6 we present five different films obtained by using the spin coating speed steps as described in Section 2 and Figure 2. Three of the samples presented are neat PVDF films and two of them are composite systems. These two latter are obtained by equal elaboration parameters (1:3 ratio of DMF/acetone at 20 °C) and contain 1 wt % of NPs amount respectively functionalized (black line pattern in Figure 6) and non-functionalized (red line pattern in Figure 6). The three neat PVDF films are obtained by the same spin coating process (1:3 ratio of DMF/acetone at 20 °C) on Si (see pink pattern in Figure 6) and on Al/Si substrates (see green pattern in Figure 6). Also we present an elaborated film on Si with different DMF-acetone ratio (1:2 ratio of DMF/acetone at 20 °C) (see blue pattern in Figure 6).

The WAXS experiment is not easy to performed, because the PVDF films thickness is very low, around 100 nm, so the diffracted intensity is too low. WAXS spectra were recorded on a selection of representative samples (see Figure 6). Although, spectra are noisy, we can identify diffraction lines associated with the PVDF crystalline phases. WAXS spectra acquired on Si and Al/Si seem to be different. Four diffraction lines, at around 18°, 18.5°, 20°, and 27°, are observed for PVDF film on Si. They are respectively indexed as the d_100_ = 4.96 Å, d_020_ = 4.82 Å, d_110_ = 4.41 Å, and d_021_ = 3.33 Å interplanar distances of the α crystalline phase. In the case of the PVDF film on Al/Si substrate, except for the two sharp peaks (♦) attributed to the substrate, only one broad peak is clearly observed at 20.7° attributable to the β or/and γ phase. The fact that the two lines around 18–18.5°are not detected suggests the absence of the α phase in this case. For the γ phase, two weak lines at around 22.5° (d_111_ = 3.95 Å) and 26.5° (d_022_ = 3.36 Å) should be observed in order to differentiate this phase from the β one [20,22,35]. As the WAXS spectra intensity is low, it is difficult to discriminate these two phases in our spectra. We can thus conclude that only for the PVDF film deposited on the Al/Si substrate, WAXS measurements clearly show β or/and γ phase. Concerning the WAXS spectra recorded on the composite films, the main diffraction lines observed are related to the spinel Ni_0.5_Zn_0.5_Fe_2_O_4_ nanoparticles [46] (see Supplementary Materials, Section S2 for more details on the NPs XRD spectrum). Excepting the Bragg peaks, a small broad peak is observed around 20.5°, may be again from the PVDF β or/and γ phase. More details are reported in the section discussion to analyze our interpretation. 

### 3.3. Infrared Transmittance of Neat and Composite PVDF Films on Different Substrates

**Figure 7:**
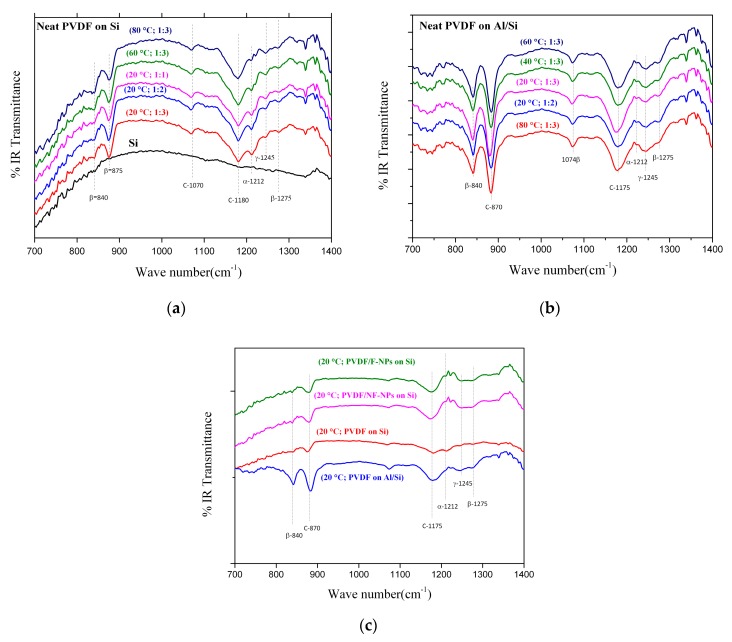
(**a**) IR transmittance of neat PVDF thin films on Si substrate with different elaboration parameters detailed in parenthesis (temperature; DMF/acetone ratio); (**b**) IR transmittance of neat PVDF thin films on Al/Si substrate with different elaboration parameters detailed in parenthesis (temperature; DMF/acetone ratio); (**c**) comparison between neat PVDF thin films on Si and Al/Si and composite thin films obtained by equal conditions of elaboration with functionalized (F-NPs) nanoparticles and non-functionalized ones (NF-NPs).

In the previous paragraph, the broad reflections observed in the X-ray diffraction patterns, lead to a confusion among the γ and β phase amount so that we decided to perform infrared spectroscopy measurements. IR is a complementary technique compared to the XRD one that can be useful to distinguish between the various phases in which the PVDF can crystallize. Indeed, the crystalline structures of PVDF depend on the molecular chain conformation, α (TGTG’), β (TTTT), and γ (T_3_GT_3_G’). As a matter of fact, in the near infrared region, each of the PVDF phases shows its own absorptions in the detectable transmittance. The intensity and the presence of these absorptions (indexed in Figure 7 from a comparison with the literature [35,47,48,49,50]) depend on the amount of the absorbent phase.

Figure 7a shows the FTIR spectra of the elaborated neat PVDF thin films on silicon substrates and Figure 7b of the correspondent elaborated neat PVDF thin films on silicon covered by aluminum. Although all these results show common absorption peaks for both substrates, in the case of Al/Si we can clearly identify by comparison with the literature a clear majority of the electro-active phases (β and γ) (Figure 7a,b), no matter the substrate temperature (20 °C to 80 °C) or the solution mixture (i.e., DMF/acetone ratio from 1:1 to 1:3). In the case of silicon substrate, it is more difficult to conclude due to the substrate absorption (black line—Figure 7a) reducing the absorption peaks intensity of the PVDF. Before going into details of the observed peaks and their identification, it is important to underline that a baseline of the spectrometer has been recorded at room temperature and subtracted from the corresponding spectrum using the spectrometer software. We also recorded the substrate transmittance in order to verify a possible artefact from the substrates absorption. Putting in comparison IR spectra in Figure 7a,b, it is possible to notice that the Si substrate (black line in Figure 7a) has a very broad absorption for wave lengths smaller than 840 cm^−1^ which prevents the correct observation of the absorption band located at exactly 840 cm^−1^, attributed to the TTT conformations existing in the β phase and also in the γ phase [35,47,48,49,50]. This band is, in fact, remarkably visible in the twin-films deposited on Al/Si substrates where this broad absorption is absent thank to the good reflectivity properties of the aluminum layer within all the wavelength range. Considering the small thickness of the film, in fact, it is reasonable to imagine that the IR light crosses all the film and is than reflected by the substrate. The second band that characterizes the β-phase is the one at 1275 cm^−1^ which is less marked but still visible for all the samples. The γ-phase is characterized by a band at 1245 cm^−1^ in addition to the one at 840 cm^−1^. This 1245 cm^−1^ band is clearly observable for the neat PVDF films on Al/Si substrate. Only one band identifying the non-polar α phase is visible in our IR spectra and located at 1212 cm^−1^, this band is mostly observed for the neat PVDF films on Si substrate. It also should be noticed that this band appears as a shoulder of a more intense peak at 1175 cm^−1^ which, together with the one at 1070 cm^−1^, has been recently [50] identified as the vibrations of the carbon–carbon skeleton in the polymer. The α phase is identified in neat PVDF films on Si substrate, while β and γ phases coexist in films on Al/Si substrate.

All these features lay true in the case of Figure 7c where neat PVDF films deposited on Si and Al/Si substrates are presented in comparison with composite PVDF films including functionalized nanoparticles (F-PVDF) and non-functionalized ones (NF-PVDF). In the composite films, the two bands at 1245 cm^−1^ and 1275 cm^−1^ are observed indicating the coexistence of the β and γ phases in these films.

### 3.4. Magnetic Properties of the Composite Thin Films

**Figure 8:**
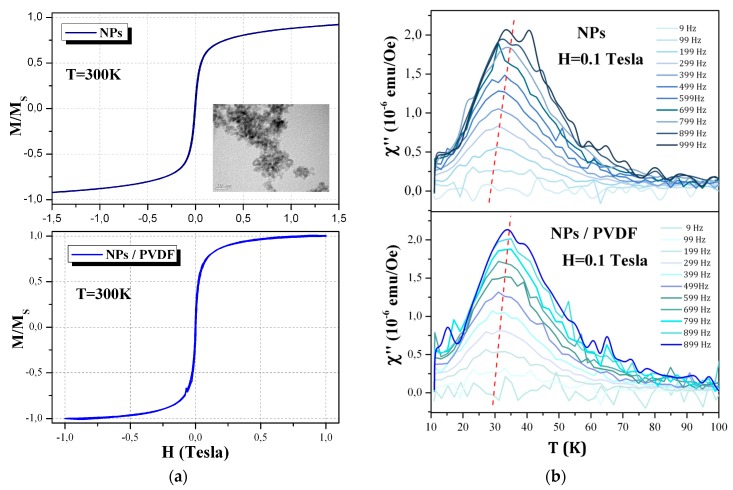
(**a**-top) hysteresis cycle at room temperature of Co-based nano-inclusions (inset: TEM image of the nanoparticles) and (**a**-bottom) composite film containing 1 wt % of functionalized nano-inclusions; (**b**-top) magnetic susceptibility as function of temperature for Co-based nano-inclusions and (**b**-bottom) composite film containing 1 wt % of functionalized nano-inclusions.

As already mentioned in a previous section, part of our optimization aimed to obtain an efficient artificial coupling between the electro-active phase of the organic thin film and the standard inorganic ferromagnetic nanoparticles. Thus concomitant electro-active and ferromagnetic properties at room temperature are important to be verified. The previous section was dedicated to the first properties while, in this section, we present the second ones. Figure 8a shows the static magnetic properties of the nanoparticles before and after their integration in the PVDF solution at room temperature. Magnetization as a function of the magnetic field applied (i.e., hysteresis cycle) shows the expected peculiar magnetic behavior at room temperature for both samples. Figure 8b shows the susceptibility behavior as function of temperature again for the nanoparticles before and after their integration in the PVDF solution. This low frequency behavior (10 Hz up to 1000 Hz) shows that the slow dynamic of the magnetic nanoparticles is preserved after their functionalization and their dispersion into the PVDF solution.

### 3.5. Micro-Structuration of the PVDF Thin Films

**Figure 9:**
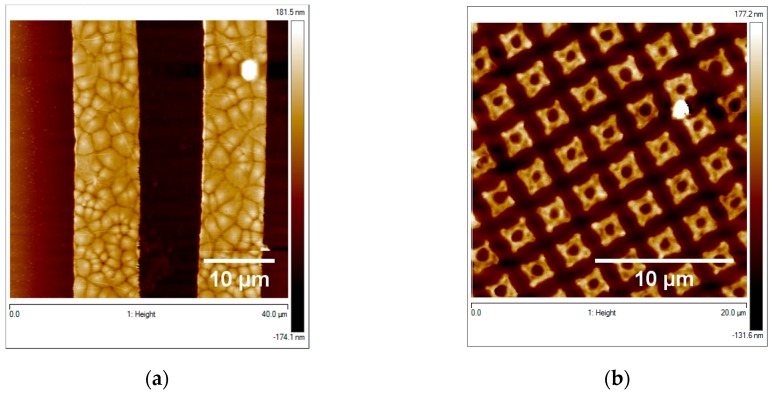
(**a**) 40 × 40 µm AFM micrograph of the 10 µm micro lines of PVDF; (**b**) 20 µm AFM micrograph of the 2 µm dimeter squared-rings.

As an example of the possibility to use structuration process from the silicon technology, PVDF thin films were micro-patterned using standard lithography and etching process. Micro lines (width of 10 µm) as well as the squared-rings patterns were first defined in the AZ1505 commercial resist spin coated on top of the PVDF thin film by using a mask aligner and a 10 s development in the commercial MF-319 developer. The patterns in the resist were then transferred through the total thickness of the PVDF thin film using RIE etching process. RIE etching parameters were defined looking into the literature for PVDF RIE etching [49]: the chamber pressure was 150 mTorr, CHF3 flow rate was 48 sccm, and RF power of 150 W. The result of both patterns transfered is presented here below in Figure 9a,b. Very well-defined PVDF lines of 10 µm width, appear on the AFM images over a big surface of the sample (more than 50 µm).

## 4. Discussion

As reported in the introduction section, the spin coating process allows controlling the β phase content, the thickness and the morphology of PVDF film in a good reproducible way [33,35,50]. All the previous results, as already explained in the introduction (see Section 1), bring up the question of either is possible to elaborate by an easy-to-transfer technique both neat PVDF thin films as well as the composite ones showing low roughness (lower than 10 nm), thickness smaller than 100 nm, and homogeneity over a large surface on a silicon-based conductive substrates. We remind here, that all these characteristics are mandatory in order to use these polymer-based films for the next generation of multifunctional (i.e., magnetoelectric) microelectronics based on the standard silicon technology. To this purpose, we decided to optimize the spin coating process by varying the speed steps, the DMF/acetone ratio of the solution and the substrate temperature. Our results show that a two-steps spinning process allow the formation of continuous and low rough thin films (see Section 3.1) whose proportion of β and γ phase is easily stabilized by the use of Al/Si substrate as well as by incorporation of functionalized nano-inclusions at room temperature. AFM images as well as the roughness and profilometer analysis show that continuous film of mean thickness of 85 nm with mean roughness between 8 and 10 nm are obtained at room temperature (20 °C). X-ray diffraction and IR results (see Section 3.2 and Section 3.3) allow concluding that we were able to obtain neat PVDF films with a clear presence of both electro-active phases (β and γ) whose X-ray peaks as well as IR absorption are better defined than the one of the α phase. Although this phase is still present in our films and thus introduces a non-electro-active percentage of matter to our samples, it diffracts and absorbs less than the electro-active ones. Our results also show the expected improvement of the β and/or γ over α phase ratio on the X-ray reflections for the composite films thanks to the nano-inclusions introduction in the PVDF solution (see in Figure 6 the disappearance of the α Bragg peaks). This is confirmed by the IR transmittance results, with the decrease of the α-phase band at 1212 cm^−1^ and the appearance of the two bands at 1245 cm^−1^ and 1275 cm^−1^ respectively associated to the γ and β phases (see Figure 7), denoting the presence of a higher amount of these latter phases. These observations confirm the optimization of the nucleation by the optimized spin coating process of the electro-active phase content in all our samples. As reported in previous sections, adding nano-fillers into the PVDF matrix has not only a technology goal of artificially coupling two different functionalities in the same nanostructures (i.e., piezoelectric and ferromagnetic), but comes basically from the idea that the nano-objects can promote the nucleation of a suitable electro-active phase. The enhancement of the β phase by using nano-fillers depends on the types of nano-inclusions as well as from the charges on their surface [25,30,31,32,33,34,35,48,50]. The interaction between the electric charged surface of the nano-inclusions and the bonds of PVDF chains has already been proposed as a key factor for increasing the nucleation of the β phase of the polymer [8,24,25,27,28,29,30,31,32]. Indeed, the presence of nano-fillers in a PVDF polymer matrix directly affects the charge distribution in the PVDF and could be also in charge of the same effect on the inorganic nano-inclusions. It is extremely important, so far, to measure the effect of this interface (functionalized or not) on the ferromagnetic properties of the nanoparticles. As reported in the materials section, we decided to use, as nano-inclusions, isotropic nanoparticles of Ni_0.5_Zn_0.5_Fe_2_O_4_ (NZFO-NPs). The morphology and the magnetic properties of our NZFO-NPs were characterized respectively by transmission electron microscopy and magnetometry techniques (see Figure 8). The size distribution of the NPs was found uniform and the average diameter was found to be 5 nm and the saturation magnetization value at 5 K reaches 63 emu/g, which is close to the expected value [46]. At room temperature, the magnetic remanence (H = 0 Tesla) as well as the coercive field (M/Ms = 0) is negligible as expected for nanometric NPs exhibiting superparamagnetic behavior (see Figure 8a top graph). This behavior is confirmed by the NPs embedded into the matrix and deposited on the Si substrate (see Figure 8a bottom graph). We can notice that in this latter case the saturation field (where M/Ms = 1 and NPs are fully aligned along the field) is slightly lower than in the case of the nanoparticles (Figure 8 top graph). It is worth noticing that ferromagnetic nanoparticles act like strong nano-magnets, thus as soon as they are sufficiently nearby (closer than 10 nm), they attract each other. Once they agglomerate, it is very difficult to separate them. By functionalizing them we also avoid this agglomeration effect and thus we decrease the magnetic interaction effects. The result is that the nanoparticles are easier to order while included (after the functionalization) in the PVDF matrix (i.e., saturation magnetization is reached faster at lower magnetic field as reported previously). To confirm this effect is coming from a better dispersion of the NPs, we performed alternative susceptibility measurements showing the slow expected dynamic for Fe-based nanometric nanoparticles. As a result of their intrinsic magnetic properties (i.e., magnetic anisotropy) the critical temperature shift observed at low temperatures (see Figure 8b top and bottom graphs) is slower in the case of the nano-inclusions embedded in the PVDF matrix. In fact, the rate of the critical temperature shifts (red dotted line in Figure 8b bottom graph) is higher in the case of functionalized NPs embedded in PVDF films. This puts again in evidence that less interactive NPs present a faster magnetic dynamic than the agglomerate ones. All these observations lead us to affirm that our composite PVDF-based thin films concomitantly present at room temperature a majority of the electro-active phase and the optimized static magnetic behavior expected for well-dispersed isotropic ferromagnetic/superparamagnetic nanoparticles.

To go further on our optimization elaboration and to demonstrate the feasibility of basic devices based on this optimized PVDF thin films, we decided to micro-structure them. Two approaches can be considered for the micro or nano-structuration of a material: the ‘top down’ or the ‘bottom up’ approach. The point of view of this study is to develop a simple process adapted to conventional silicon technology; therefore, only ‘top down’ technics were considered. Very few papers have reported the micro or nano-structuration of PVDF thin films via top down approach as a direct photo-etching using synchrotron X-rays [51] or a laser plasma UV [52]. However, those techniques are not well adapted for industrial process. Nano-imprint lithography was also investigated with few issues such as high aspect ratio features [53]. The process presented here to fabricate micro-patterns of PVDF, is a photolithography technique combined with RIE etching. This technique was recently reported for etching PVDF thin films. Miki et al. [49] and Jiang et al. [54] experimented different gas mixture and etching conditions, on which we based our RIE process. Fluorine chemistry plasma (CHF_3_) with a flow rate of 48 sccm was perfectly adapted in our case. The total thickness of PVDF thin film (around 100 nm) was completely etched through the photoresist mask with a thickness of 500 nm. The well-defined 10 µm wide stripes as well as the regular squared-rings of 2 µm diameter of thin PVDF film obtained with this simple process, indicate the possibility to investigate process with smaller patterns up to the nanoscale in the near future. Last but not least, the micrometric spherulites clearly observed in Figure 9a, invoke the optimized PVDF morphology in Figure 2, Figure 3 and Figure 4 and thus strongly support the fact that the “top down” technique we used is non-destructive for the polymer properties. Our results open the way for future industrial use of the one-dimensional-patterned device in the new multifunctional microelectronics.

## 5. Conclusions

In conclusion, we have investigated the optimization process of poly(vinylidene fluoride) (PVDF) thin film deposition with a well-controlled thickness, roughness, and nano-inclusions amount. Spin-coating deposition technique performed on various substrates (glass, silicon, and Al/silicon) allows realizing neat-PVDF and composite PVDF-based film with mean thickness of 90 nm, continuous over a large substrate area (4 × 4 mm) and smooth (typical roughness lower than 10 nm). We show that this is possible by properly adjusting the concentration of the PVDF solution as well as the spin rate and the substrate temperature of the elaboration process. Concomitant electro-active phase optimization and ferromagnetic/superparamagnetic properties have been observed at room temperature. We demonstrate, in the case of the neat PVDF films, the clear presence of both electro-active phases (β and γ) showing X-ray diffraction peaks as well as IR absorption band, better defined than the one of the α phase. Our results also show the expected improvement of the β (and/or γ) over α phase ratio for the composite films thanks to the nano-inclusions introduction. Last but not least, we also demonstrate the easy feasibility of PVDF micro-lines as well as squared-ring-based structures by micro-patterning optical lithography combined with plasma etching. This opens the way for the easy-to-transfer silicon technology for polymer-based composite devices.

## Figures and Tables

**Table 1:** Morphology characteristics of the PVDF thin films with different volume ratio of DMF/acetone.

DMF/Acetone Volume Ratio	Homogeneity	Mean Roughness (Ra)	Mean Thickness
1:1	non-continuous	7 nm	113 nm
1:2	continuous	12 nm	101 nm
1:3	continuous	10 nm	90 nm

**Table 2:** Morphology characteristics of the PVDF thin films as function of the substrate temperature and the substrate type.

Substrate Temperature (±5%)	Substrate Type	Homogeneity	Mean Roughness (Ra)	Mean Thickness
RT (20 ± 1) °C	doped silicon Al/silicon	continuous continuous	10 nm 9 nm	85 nm 101 nm
(40 ± 2) °C	doped silicon Al/silicon	continuous continuous	6 nm 11 nm	70 nm 92 nm
(60 ± 3) °C	doped silicon Al/silicon	continuous continuous	4 nm 12 nm	90 nm 99 nm
(80 ± 4) °C	doped silicon Al/silicon	non-continuous non-continuous	3 nm 3 nm	83 nm 79 nm

**Table 3:** Morphology characteristics of the composite PVDF thin films as function of the NPs concentration and their functionalization.

NPs wt %	Categories of NPs	Homogeneity	Mean Roughness (Ra)	Mean Thickness
0.5	Non-functionalized Functionalized	Continuous Continuous	4 nm 6 nm	76 nm 80 nm
1	Non-functionalized Functionalized	Continuous Continuous	13 nm 14 nm	121 nm 97 nm

**Figure A1:**
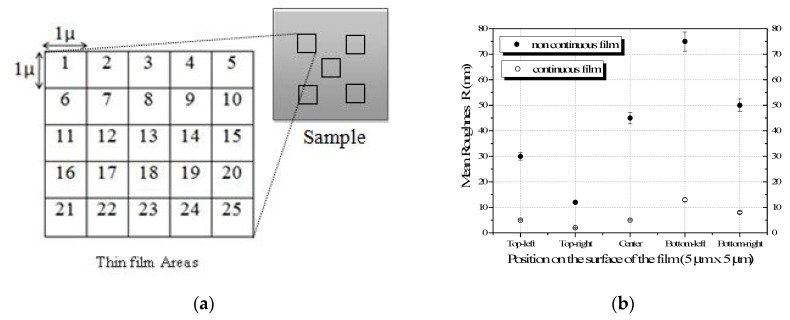
(**a**) Scheme of the 5 × 5 µm AFM micrograph analysis. Each sample surface imaged by AFM standard microscope, has been divided in five zones (see the right top of the figure (a)) and each of these zones has been analyzed in order to obtain a roughness value for each 1 × 1 µm square reported in the scheme. (**b**) The mean roughness value of each zone is here reported as function of the five different zones analyzed for two different kinds of samples (continuous and not continuous).

## Supplementary Materials

The following are available online at https://www.mdpi.com/1996-1944/13/6/1342/s1, Figure S1: WAXS spectra of Ni_0.5_Zn_0.5_Fe_2_O_4_ nanoparticles (a) recorded with the λ_Co_ radiation and (b) modified for the λ_Cu_ radiation. The Bragg peaks indexed with ‘∗’ are those identified in the Figure 6 for the composite samples, Figure S2: (left) spin-coater set-up; (right) spin-coater controller, Table S1: Proportions of the PVDF composite solutions.

## Appendix A: Analysis of the Thin Film Roughness

The topography of thin film was measured at five different positions over the surface of each film. In order to be sure of the homogeneity of the deposition process, we analyzed the surface roughness of the film at the top left, top right, center, bottom left, bottom right positions respectively (see in Figure A1 the 5 regions selected on the sample surface (grey area)). For a reasonable statistical approach, the overall topography of one position is determined by dividing the area into 25 regions (see in Figure A1 the thin film areas), followed by defining the roughness of each region. Then, the final value is obtained by the mean of the obtained roughness’s values over the 25 regions. This analysis has been done by using the Nanoscope program.
