# Supplementary material for: Spin Coating and Micro-Patterning Optimization of Composite Thin Films Based on PVDF
Source: Materials (Basel). 2020 Mar 16;13(6):1342. doi: 10.3390/ma13061342 (PMC7143455; doi:10.3390/ma13061342)
Supplement: Supplementary file 1 [file materials-13-01342-s001.pdf]

*materials*

*Supplementary*

# Spin Coating and Micro-Patterning Optimization of Composite Thin Films Based on PVDF

**Anh Ngoc Nguyen [1,2], Jeanne Solard [3], Huyen Thi Thanh Nong [1,4], Chirine Ben Osman [5], Andres Gomez [6], Valérie Bockelée [1], Sylvie Tencé-Girault [7], Frédéric Schoenstein [1], Maite Simón-Sorbed [6], Anna Esther Carrillo [6], and Silvana Mercone [1,*]**

[1] Laboratoire de Sciences des Procédés et des Matériaux (LSPM-CNRS UPR-3407), Université Sorbonne Paris Nord (USPN), 93430 Villetaneuse, France; anhnn@hus.edu.vn (A.N.N.); thanhhuyen.vltn@gmail.com (H.T.T.N.); valerie.bockelee@lspm.cnrs.fr (V.B.); frederic.schoenstein@univ-paris13.fr (F.S.)
[2] Institute of Materials Science, Vietnam Academy of Science and Technology, Cau Giay Distr., Hanoi, Vietnam
[3] Laboratoire de Physique des Lasers (LPL-CNRS UMR-7538), Université Sorbonne Paris Nord (USPN), 93430 Villetaneuse, France; jeanne.solard@univ-paris13.fr
[4] Institute Jean Lamour, UMR 7198 CNRS - Lorraine University Campus Artem, 54000 Nancy, France
[5] R&I Silica Synthesis Engineer, SOLVAY, 92400 Courbevoie, France; ch.benosman@gmail.com
[6] Instituto de Ciencia de Materiales de Barcelona (ICMAB-CSIC), Campus UAB, 08193 Bellaterra, Spain; agomez@icmab.es (A.G.); msimon@icmab.es (M.S.-S.); anaesther@icmab.es (A.E.C.)
[7] PIMM, Arts et Metiers Institute of Technology, CNRS, Cnam, HESAM University, 151 Boulevard de l'Hopital, 75013 Paris, France; Sylvie.GIRAULT@ensam.eu
* Correspondence: silvana.mercone@univ-paris13.fr

Received: 24 January 2020; Accepted: 12 March 2020; Published: 16 March 2020

## 1. $Ni_{0.5}Zn_{0.5}Fe_2O_4$ Nanoparticles Synthesis

$Ni_{0.5}Zn_{0.5}Fe_2O_4$ nanoparticles have been synthesized by forced hydrolysis in polyols. This soft chemistry method has been developed in 1980 by Fièvet et al. [38] and has been widely optimized for $MFe_2O_4$ (M = Co, Zn, Ni...) ferrites nanoparticles synthesis [39–43]. Two types of chemical reactions can be carried out in polyol medium: reduction and hydrolysis. The competition between these reactions is essentially governed by the rate of hydrolysis H of the reaction medium defined as being the ratio between the number of moles of water and the number of moles of the metal ($H = nH_2O/nM$). The absence of water leads to the metal, a moderate rate leads to the oxide and a high rate leads to the hydroxide. This process is particularly well suited for the elaboration of nanoparticles presenting well-controlled size and morphology since it allows a good control of the nucleation and growth stages.

For the synthesis of $Ni_{0.5}Zn_{0.5}Fe_2O_4$ nanoparticles, ionic salts: iron (III) chloride hexahydrate, nickel (II) acetate tetrahydrate, zinc (II) acetate dihydrate, and anhydrous sodium acetate were dissolved in stochiometric ratio in 1,2-propanediol (1 L) and heated (temperature ramp: 4 °C/min) while being mechanically stirred. The polyol acts as a solvent for the salt precursor (high permittivity). It allows to carry out hydrolysis reactions at high temperature up to the boiling point of the polyol and it plays the role of crystal growth media due to its supernatant properties [40]. The total metal ion concentration was 0.3 $mol \cdot L^{-1}$ and the hydrolysis ratio, was fixed by the amount of water in the precursor salts. The sodium acetate ratio defined as $nNaOAc/nM$ was set at 3. The mixture was refluxed (at 150 °C) for 3 h under mechanical stirring (300 tr/min). After cooling to room temperature, the nanoparticles were separated from the supernatant by centrifugation, washed three times with ethanol, then only one time by acetone and deionized water. Then, the ferrite phase was dried in air at 50 °C. Finally, the as-prepared powders have been thermally treated during 6 h at 300 °C under air to remove the remaining adsorbed organic species. The purity of the powder is controlled by XRD analysis as follows.

materials MDPI

## 2. X-ray Diffraction of Nanoparticles

For the $Ni_{0.5}$ $Zn_{0.5}Fe_2O_4$ nanoparticles study, we used the XRD Equinox 1000 (INEL) diffractometer with a Co-target K$\alpha$ radiation ($\lambda$ = 1.7890 Å). The recorded spectra is reported Figure 1a.

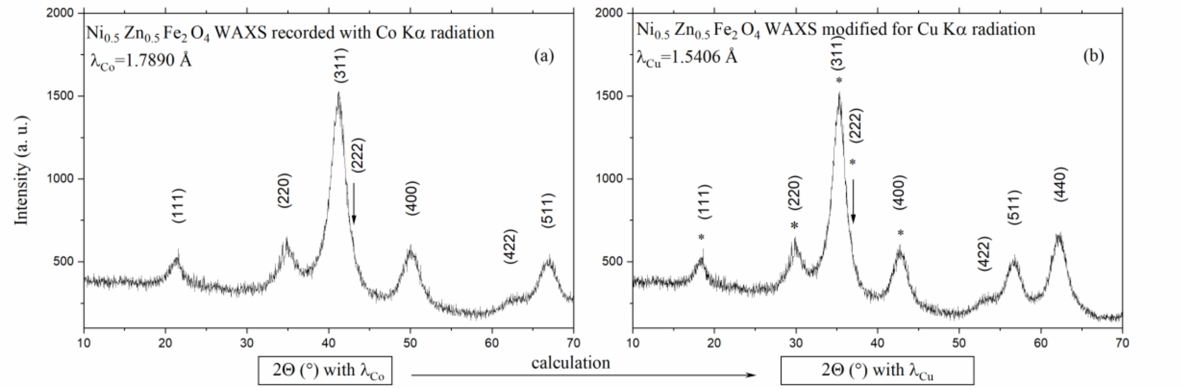


**Figure S1.** WAXS spectra of $Ni_{0.5}Zn_{0.5}Fe_2O_4$ nanoparticles (**a**) recorded with the $\lambda_{Co}$ radiation and (**b**) modified for the $\lambda_{Cu}$ radiation. The Bragg peaks indexed with '*' are those identified in the Figure 6 for the composite samples.

WAXS experiments on nanoparticles are carried out with the Co-Kα radiation ($\lambda$ = 1.7890 Å), while those on PVDF films are recorded with the Cu-Kα radiation ($\lambda$ = 1.5406 Å). To modify the 2θ scale for the Cu radiation, we used the Bragg law for the two radiations

$$2d_{hkl}\sin\theta_{hkl}^{Co} = \lambda_{Co} \text{ and } 2d_{hkl}\sin\theta_{hkl}^{Cu} = \lambda_{Cu}$$

By eliminating the distances $d_{hkl}$, which are characteristic of the sample, and does not depend on the wavelength, we obtain

$$sin\theta^{Cu} = \frac{\lambda_{Cu}}{\lambda_{Co}}\sin\theta^{Co}$$

Using this relation, we calculate the WAXS spectra Figure 1b. The indexation of the Bragg peaks with the (hkl) planes is deduced from literature [46]. The Bragg peaks indexed with «*» in Figure 1b are those detected in the composite films (Figure 6 of the main text).

## 3. Functionalization Process of Nanoparticles

The functionalization has been carried out by following the procedure reported in Ben Osman et al. [26] and Liu et al. [25].

The surface of $Ni_{0.5}Zn_{0.5}Fe_2O_4$ NPs were functionalized by a hydrophobic fluorinated phosphonic acid named 1H,1H,2H,2H perfluorooctane phosphonic acid (PFOP) in order to increase their miscibility and enhance their dispersion in PVDF solution in DMF/acetone.

100 mg of nanoparticles have been dispersed in 100 mL of tetrahydrofuran (THF) and sonicated for 30 min. Then, 32 mg of 1H,1H,2H,2H perfluorooctane phosphonic acid (PFOP) have been introduced and the mixture has been sonicated for 60 min. Finally, the NP-PFOP has been precipitated by acetone, recovered by magnetic decantation, washed several times by centrifugation with a mixture of THF-acetone (1/2 v/v). Before their introduction in PVDF, the functionalized nanoparticles have been dried at 60 °C for 24 h.

## 4. Clean Room Set Up

Our spin process procedure consists of dispensing a small amount of PVDF solution with a micropipette onto the substrate which is hold with vacuum on the spin-coater chuck. The substrate

is then rotated at high speed. The spin speed is programmed via a controller allowing the variation of the speed and the time of the different stages. We can thus create our own programs.

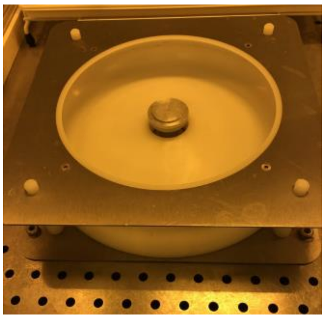

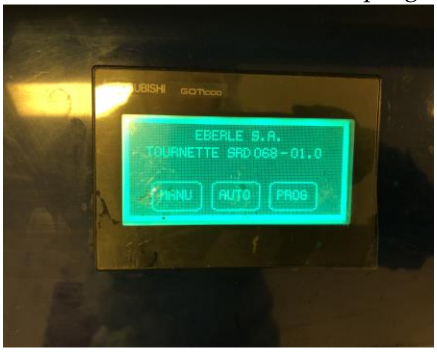


**Figure S2.** Spin-coater set-up (left); spin-coater controller (right).

In order to realize the PVDf films reported in the main text of the article at different temperature, substrates were heated in the oven for 10 min in order to reach the thermal equilibrium. The calibration of the thermal equilibrium between 30 °C and 100 °C of the oven has been done by using a thermocouple (K type). It takes 5 seconds to begin spin coating after removing the substrate from the oven and put it on the spin coater. As reported in Section 3 of the main text, the realization of the films by process 4 (see Figure 2 of the main text), took typically 72 s. After the process we observed a decreasing of the substrate temperature varying between 2 °C and 5 °C. This corresponds to a maximum variation of the reported temperatures never exceeding the 5%. Proportions of the PVDF, nanoparticles and DMF/acetone solution used for the production of the composite films are reported in the following table:

**Table S1.** Proportions of the PVDF composite solutions

| NPs wt.% | Categories of NPs | Nanoparticles | PVDF | Acetone | DMF |
|---|---|---|---|---|---|
| 0.5% | Functionalized | 41.7 mg | 79.5 mg | 7.5 mL | 2.5 mL |
| | Non functionalized | | | | |
| 1% | Functionalized | 83.4 mg | 79.5 mg | 7.5 mL | 2.5 mL |
| | Non functionalized | | | | |
